# Tick-borne Encephalitis Virus in Horses, Austria, 2011

**DOI:** 10.3201/eid1904.121450

**Published:** 2013-04

**Authors:** James O. Rushton, Sylvie Lecollinet, Zdenek Hubálek, Petra Svobodová, Helga Lussy, Norbert Nowotny

**Affiliations:** University of Veterinary Medicine, Vienna, Austria (J.O. Rushton, H. Lussy, N. Nowotny);; Agence Nationale de Sécurité Sanitaire, Maisons-Alfort, France (S. Lecollinet);; Academy of Sciences of the Czech Republic, Brno, Czech Republic (Z. Hubálek, P. Svobodová);; Sultan Qaboos University, Muscat, Oman (N. Nowotny)

**Keywords:** tick-borne encephalitis virus, viruses, tickborne encephalitis, flavivirus, Flaviviridae, horses, prevalence, infection, vector-borne infections, Austria

## Abstract

An unexpectedly high infection rate (26.1%) of tick-borne encephalitis virus (TBEV) was identified in a herd of 257 horses of the same breed distributed among 3 federal states in Austria. Young age (p<0.001) and male sex (p = 0.001) were positively associated with infection.

Tick-borne encephalitis (TBE), which is caused by tick-borne encephalitis virus (TBEV), is a potentially fatal disease of the central nervous system, mainly in humans, but also in monkeys, dogs (*1*), and horses. Ruminants such as goats, sheep, and cattle are considered to be sporadically infected subclinically. However, they might be the source of disease in humans who consume nonpasteurized milk and milk products (*2*). TBEV-associated central nervous system disease in ruminants is rare (*3*).

TBEV occurs in natural foci and is endemic to many countries in Europe and parts of central and eastern Asia (*4*). The principal vectors for transmission are ticks of the genus *Ixodes*. Although TBEV in humans has been studied extensively, there are only a limited number of reports on TBEV in animals (*5*), especially horses. Only 2 reports were found in the German literature on the epidemiology of TBEV infection in horses (*6**,**7*), and 1 case report was found on clinical symptoms of TBE in a mare (*8*). The purpose of this study was to determine the status of TBEV infection in a large population of a single horse breed in Austria.

## The Study

Serum samples from 257 horses of the same breed that were distributed among 3 federal states in Austria were obtained in April 2011 and screened by using a commercial ELISA (ID Screen West Nile Competition ELISA Kit; IDvet, Montpellier, France) for antibodies against flaviviruses. ELISA-positive serum samples were further investigated by using virus-specific neutralization assays for the 3 flaviviruses circulating in Austria (West Nile virus [WNV], Usutu virus [USUV], and TBEV). Neutralization assays were conducted independently by 2 laboratories (*9**,**10*). Results were analyzed by using SPSS version 17 software (SPSS IBM, Armonk, NY, USA). Associations of sex, age, and location with positive results were tested by using 1-way analysis of variance and tested for significance by using the χ^2^ test. Differences in age between horses positive or negative for flaviviruses were determined by using the Student *t*-test. A p value <0.05 was considered significant for all analyses.

The study comprised 113 (44.0%) mares, 139 (54.0%) stallions, and 5 (2.0%) geldings. The mean ± SD age of horses was 8.1 ± 6.3 years (range 1–32 years). A total of 154 (59.9%) horses were boarded in Styria, 66 (25.7%) in Vienna, and 37 (14.4%) in Lower Austria and kept in various types of housing. All 3 locations are considered areas to which WNV, USUV, and TBEV are endemic. The animals were free from clinical symptoms associated with flavivirus infections. None of the horses were vaccinated with WNV and TBEV vaccines (TBEV vaccines are not licensed for use in horses).

Sixty-seven (26.1%) horses were positive for antibodies against flaviviruses by ELISA, and all 67 were positive for TBEV by virus-specific neutralization tests (Table, Appendix, wwwnc.cdc.gov/EID/article/19/4/12-1450-T1.htm). Positive results were distributed among 17 mares, 49 stallions, and 1 gelding. The difference in results between sexes was significant (p = 0.001) (Figure). The mean ± SD ages of horses positive and negative for TBEV antibodies were 5.9 ± 4.2 and 8.9 ± 6.7 years, respectively (p<0.001) (Figure). Thirty-seven positive horses were kept in Styria, 16 in Vienna, and 14 in Lower Austria. The difference in positive results for horses at the 3 locations was not significant. Low-level cross-reactivity for WNV and USUV was observed in 9 animals (Table).

**Table:** WNV-, USUV-, and TBEV-specific neutralization assay results obtained in 2 laboratories for 67 horses positive for flaviviruses by ELISA, Austria, 2011*

Horse no.	Age, y	Sex	Location	Virus strains and titers				
				WNV Is98, Laboratory 1	WNV Eg101, Laboratory 2	USUV SAAR 1776, Laboratory 1	TBEV Hypr, Laboratory 1	TBEV Hypr, Laboratory 2
1	3	Stallion	Lower Austria	<10	<10	<10	100	>40†
2	3	Stallion	Lower Austria	<10	10	<10	>100‡	>40
3	3	Stallion	Lower Austria	<10	10–20	<10	>100	>40
4	4	Stallion	Lower Austria	<10	<20§	<10	>100	>40
5	4	Stallion	Lower Austria	<10	<20§	<10	>100	>40
6	4	Stallion	Lower Austria	<10	<10	<10	>100	>40
7	4	Stallion	Lower Austria	<10	<10	<10	>100	≥40
8	5	Stallion	Lower Austria	<10	<20§	<10	>100	>40
9	5	Stallion	Lower Austria	<10	<10	<10	>100	>40
10	7	Stallion	Lower Austria	<10	<20§	<10	>100	>40
11	7	Stallion	Lower Austria	<10	<10	<10	>20¶	>40
12	8	Stallion	Lower Austria	<30§	<10	<10	>20¶	>40
13	10	Stallion	Lower Austria	<30§	<10	<30§	>20¶	>40
14	13	Stallion	Lower Austria	<10	<10	<10	>100	>40
15	1	Stallion	Styria	<10	<20§	<10	>100	>40
16	1	Stallion	Styria	<10	<10	<10	20	20
17	1	Mare	Styria	<10	<10	<10	>100	>40
18	2	Mare	Styria	<10	10	<10	100	>40
19	2	Stallion	Styria	10	<20§	10	>100	20–40
20	2	Stallion	Styria	<10	<20§	<10	>100	>40
21	2	Stallion	Styria	<10	<10	<10	>100	>40
22	2	Stallion	Styria	<10	<20§	<10	>100	>40
23	2	Stallion	Styria	<10	<10	<10	>20¶	>40
24	2	Stallion	Styria	<10	<20§	<10	>100	>40
25	2	Stallion	Styria	<10	<20§	<10	>100	>40
26	2	Stallion	Styria	<10	≤40§	<10	>20¶	≥40
27	3	Stallion	Styria	<10	<20§	<10	>100	>40
28	3	Mare	Styria	<10	10–20	<10	100	>40
29	3	Stallion	Styria	<10	<20§	<10	>100	>40
30	3	Stallion	Styria	<10	20	<10	>100	>40
31	3	Stallion	Styria	<10	<20§	<10	>100	>40
32	3	Mare	Styria	<10	10	<10	>100	>40
33	3	Stallion	Styria	<10	<10	<10	>100	>40
34	4	Stallion	Styria	<10	≤10§	<10	20	>40
35	4	Mare	Styria	<10	<10	<10	>100	>40
36	4	Stallion	Styria	<10	≤20§	<10	>100	>40
37	4	Mare	Styria	<10	20	10	>100	>40
38	4	Mare	Styria	<10	<20§	<10	>100	>40
39	4	Gelding	Styria	<10	<10	<10	>100	>40
40	4	Stallion	Styria	<10	<10	<10	>100	>40
41	5	Mare	Styria	<10	<20§	<10	>100	≥40
42	5	Mare	Styria	<10	<10	<10	>30¶	≥40
43	5	Mare	Styria	<10	<10	<10	20	20–40
44	6	Mare	Styria	10	<2§	10	100	>40
45	7	Mare	Styria	10	<10	10	>20¶	>40
46	7	Mare	Styria	<10	<10	<10	>100	>40
47	11	Mare	Styria	<10	<20§	<10	20	>40
48	11	Mare	Styria	<10	≤10§	<10	20	>40
49	11	Stallion	Styria	<10	<20§	<10	100	>40
50	13	Mare	Styria	<10	<10	<10	>30¶	>40
51	19	Mare	Styria	<10	<10	<10	>100	>40
52	5	Stallion	Vienna	<10	<10	<10	>30¶	>40
53	5	Stallion	Vienna	<10	20	<10	>30¶	>40
54	5	Stallion	Vienna	<10	<10	<10	>40¶	>40
55	6	Stallion	Vienna	<10	20	<10	>20¶	>40
56	6	Stallion	Vienna	<10	<20§	<10	>100	>40
57	6	Stallion	Vienna	<10	<20§	<10	>100	≥40
58	7	Stallion	Vienna	<10	<10	<10	>100	>40
59	7	Stallion	Vienna	<10	<10	<10	>100	>40
60	9	Stallion	Vienna	<10	<20§	<10	>100	>40
61	11	Stallion	Vienna	<10	<20§	<10	>20¶	>40
62	11	Stallion	Vienna	<10	<20§	<10	>100	>40
63	11	Stallion	Vienna	<10	10	<10	>100	>40
64	12	Stallion	Vienna	<10	20	<10	>20¶	>40
65	13	Stallion	Vienna	<10	<20§	<10	>20¶	>40
66	14	Stallion	Vienna	<10	20	<10	>20¶	>40
67	20	Stallion	Vienna	<10	<10	<10	>100	>40

*A titer ≥10 was positive for West Nile virus (WNV) Is98 and Usutu virus (USUV) SAAR 1776 strains, and a titer ≥20 was positive for WNV Eg101 and tickborne encephalitis virus (TBEV) Hypr strains. †Titrated up to a dilution of 1:40. ‡Titrated up to a dilution of 1:100. §Cytotoxic at lower dilutions. ¶Insufficient sample material to analyze higher serum dilutions.

**Figure:**
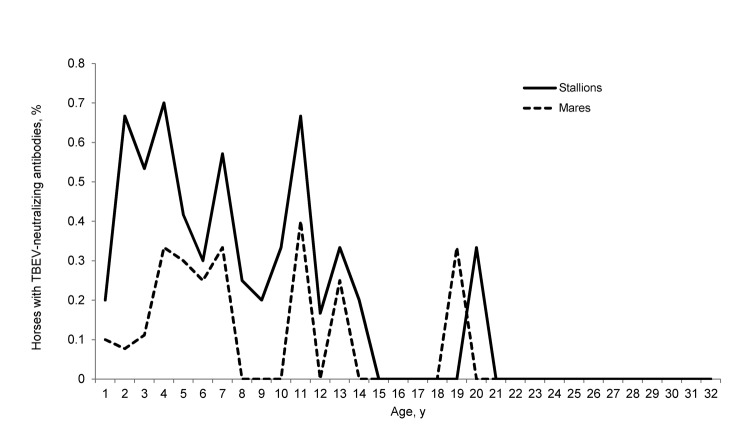
Percentages of horses seropositive for tick-borne encephalitis virus (TBEV) by age and sex, Austria, 2011. Geldings were excluded for better illustration. The difference between groups was significant for young age (p<0.001) and male sex (p = 0.001).

## Conclusions

The main findings of our study were a comparatively high seropositivity rate of 26.1%, a higher prevalence of TBEV-specific antibodies in younger horses, and a higher prevalence of TBEV-specific antibodies in stallions. We expected the horses to have subclinical infections with WNV lineage 2, which was introduced recently into central Europe (*11*), including Austria (*12*). However, it is well known that WNV IgG ELISAs show cross-reactivity with other flaviviruses, necessitating the use of virus-specific neutralization assays for identification of an etiologic flavivirus.

The population in our study had a 2-fold higher infection rate than that observed in a similar study in Austria in 1999 in a population of 468 horses (*6*). A partial explanation for this difference might be yearly fluctuating TBEV prevalence, as measured by diagnosed human infections (http://zecken.at/fsme/fsme-faelle-in-oesterreich/). In 1999, the lowest number (41) of human TBE cases was recorded in Austria (87, 79, 63, and 113 human cases were diagnosed in 2008, 2009, 2010, and 2011, respectively). A study in Germany in 2006 identified 2.9% of 240 horses with TBEV neutralizing antibodies (*7*). A more recent update on TBEV seropositivity in other animal species showed prevalence rates of 26.5% in cattle and 7.0% in sheep (*10*). This study did not detect antibodies against TBEV in 40 horses. A study on the prevalence of TBEV in dogs in Austria reported that 131 (24.0%) of 545 dogs examined had antibodies against TBEV (*13*).

We did not observe any influence of location on the likelihood of TBEV seropositivity. All 3 locations are within TBEV-endemic areas (http://zecken.at/fsme/verbreitungsgebiete/). However, none of the seropositive horses showed any clinical symptoms of an arbovirus infection at any time.

Regarding age distribution, a higher proportion of younger horses (mean age 5.9 years) had antibodies against TBEV than older horses (e.g., none of the horses 15–18 and 21–32 years of age had antibodies against TBEV). Older horses were in the same pastures as young horses. This finding contrasts with results of epidemiologic studies in cattle, in which animals ≤3 years of age showed a lower prevalence of antibodies against TBEV than did older animals (*10*). 

Tick exposure has always been high in the investigated areas, as reported in a study conducted >10 years ago, in which 52%–93% of horses were positive for antibodies against *Borrelia afzelii* by immunoblotting. Most of these horses had already been infected during their first year of age and were subsequently reinfected (*14*). Thus, older horses in our study might have been infected at a young age and showed a subsequent decrease in neutralizing antibodies below the detection limit.

The reason for the high number of seropositive stallions is unclear because stallions in the study were distributed among all 3 locations, and 33 (67.3%) of 49 were boarded in boxes (individual stable compartments that limit contact with other members of the population). Mares were kept exclusively in 1 TBEV-endemic location, mainly in pastures. However, stallions were more frequently transferred to other regions (e.g., for mating), where they might have been infected because of potentially higher tick infestation rates. It is also possible that for unknown biological reasons males are more frequently affected by ticks than females, as suggested by Perkins et al. (*15*) in a study on the yellow-necked mouse (*Apodemus flavicollis*).We plan to conduct further experiments to elucidate why ticks seem to be more attracted to male hosts than female hosts.

We observed comparatively low antibody prevalence in yearlings of both sexes, which was probably caused by decreasing, but still protective, maternally transmitted immunity. Seropositivity peaks in both sexes at 4, 7, 11, 13, and 19–20 years of age indicated infections and subsequent reinfections in certain years with higher TBEV activity (Figure).

Our study suggests that horses are prone to TBEV infection. However, they remain mostly asymptomatic. Thus, horses may be considered sentinel hosts for monitoring the spread of TBEV.
